# A Quantitative Account of the Behavioral Characteristics of Habituation: The Sometimes Opponent Processes Model of Stimulus Processing

**DOI:** 10.3389/fpsyg.2019.00504

**Published:** 2019-03-15

**Authors:** Yerco E. Uribe-Bahamonde, Sebastián A. Becerra, Fernando P. Ponce, Edgar H. Vogel

**Affiliations:** University of Talca, Talca, Chile

**Keywords:** habituation, priming, SOP, stimulus processing, stimulus intensity

## Abstract

Habituation is defined as a decline in responding to a repeated stimulus. After more than 80 years of research, there is an enduring consensus among researchers on the existence of 9–10 behavioral regularities or parameters of habituation. There is no similar agreement, however, on the best approach to explain these facts. In this paper, we demonstrate that the Sometimes Opponent Processes (SOP) model of stimulus processing accurately describes all of these regularities. This model was proposed by Allan Wagner as a quantitative elaboration of priming theory, which states that the processing of a stimulus, and therefore its capacity to provoke its response, depends inversely on the degree to which the stimulus is pre-represented in short-term memory. Using computer simulations, we show that all the facts involving within-session effects or short-term habituation might be the result of priming from recent presentations of the stimulus (self-generated priming). The characteristics involving between-sessions effects or long-term habituation would result from the retrieval of the representation of the stimulus from memory by the associated context (associatively generated priming).

## Introduction

The predominant consequence of stimulus repetition is a systematic decrease in the frequency or amplitude of the response to the stimulus. When it is proved that this decrement is not caused by physiological changes at the sensory or motor levels, it is inferred that a learning phenomenon, known as habituation, has occurred. Habituation has been experimentally studied since the early twentieth century (Humphrey, 1933; Prosser and Hunter, 1936; Harris, 1943) and its core behavioral regularities were soon compiled by Thompson and Spencer (1966) and Groves and Thompson (1970) into a list of nine characteristics or parameters of habituation. This list has remained relatively uncontroversial and has oriented most of the research in the field over the years. Indeed, 40 years after the publication of these characteristics, a group of recognized researchers in the area gathered in a symposium where one of the goals was to revisit the empirical status of these features. With minor amendments and the addition of one characteristic, the conclusion of the symposium was essentially confirmatory (Rankin et al., 2009; Thompson, 2009).

No similar agreement has been reached, however, concerning theories of habituation. Three approaches have dominated the field over the years: Groves and Thompson’s (1970) dual process theory, Sokolov’s (1960) comparator theory, and Wagner’s (1981) Sometimes Opponents Processes model (SOP). Although there is not a plethora of choices, these theories have not been systematically compared. This is likely due, in part, to the fact that they differ in their level of formalization and emphasis on different subsets of empirical data. Certainly, these theories have each their respective merits (see, Mackintosh, 1987; Hall, 1991; Siddle, 1991 for critical reviews); but, in our opinion, only SOP is formulated with sufficient quantitative detail to make relatively unambiguous descriptions of a broad spectrum of phenomena and testable predictions.

In an early chapter, Whitlow and Wagner (1984) exposed in detail the potential of SOP on this topic. However, their analysis was more conceptual than quantitative. Alternatively, Donegan and Wagner (1987) and Wagner and Vogel (2010) presented a quantitative analysis of SOP, but they focused primarily on the kind of response decrement that might be attributed to associative factors. In this paper, we attempt to complement these efforts by evaluating the quantitative performance of the model on a relatively larger set of phenomena. We also propose possible instantiations of some mechanisms that were left unspecified in previous formulations of SOP.

In the first part, we briefly describe the major principles of SOP emphasizing those more closely related to habituation. We show the theoretical mechanisms by which the habituation of any stimulus can be understood as the result of two types of memorial priming: a transient memorial effect due to recent exposure to the stimulus (Davis, 1970; Whitlow, 1975; Vogel and Wagner, 2005) and a more persisting memorial effect due to the context carrying a relatively stable association with the habituated stimulus (e.g., Jordan et al., 2000). Then, we proceed to demonstrate, by computer simulations, how these mechanisms account for the 10 parameters of habituation accorded by Rankin et al. (2009). In the last part, we discuss the potential of the model to embrace the related phenomenon of sensitization, and we comment on the limitations of our current analysis.

## The SOP Model

**Figure 1:**
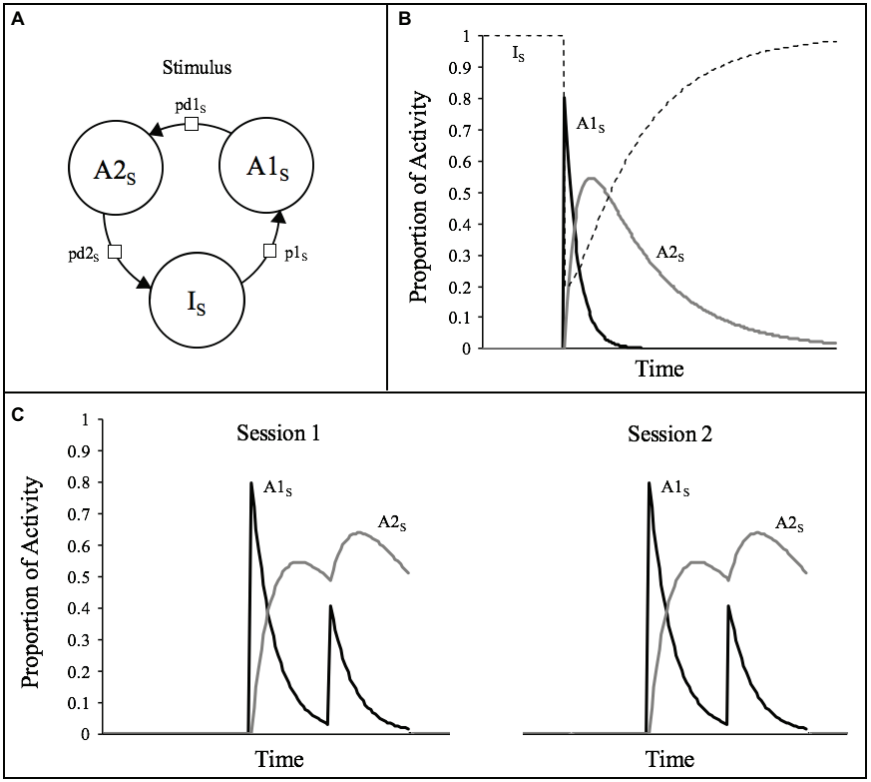
**(A)** The three possible activity states for a stimulus representation assumed by SOP. **(B)** Courses of activity for a typical stimulus (s; duration = 1 moment, *p*1_S_ = 0.8, *pd*1_S_ = 0.1, *pd*2_S_ = 0.02) according to SOP rules of activity. **(C)** Simulations of the theoretical processes involved in two training sessions of two presentations of a 1-moment stimulus.

The SOP model is described in more detail elsewhere (e.g., Wagner, 1981; Mazur and Wagner, 1982; Vogel et al., 2018), so we present only its essentials here. As shown in Figure 1A, the model states that the representation of any stimulus (i.e., “s”) comprises a large set of elements that can be in one of three states of activity: inactive (I_s_), primary activity (A1_s_), and secondary activity (A2_s_). Upon presentation of the stimulus, a proportion of the inactive elements are promoted to the A1_s_ state according to the probability *p*1_s_, which might be taken to be a function of the intensity of the stimulus. Once in the A1_s_ state, the elements decay, first to the A2_s_ state, with probability *pd*1_s_, and then back to inactivity with probability *pd*2_s_, where they remain unless a new presentation of the stimulus occurs. Thus, the momentary theoretical processing of the stimulus can be characterized by the proportion of elements in each of the three states, that is, by the vector (PI, PA1, PA2) where PI + PA1 + PA2 = 1 (Donegan and Wagner, 1987). It is assumed that the primary response to the stimulus is a function of P_A1_ and that P_A2_ might be either behaviorally silent or add to or oppose the primary response.

Let us consider the example depicted in Figure 1B, which exemplifies the momentary distribution of elements across the three states of activity over time after a single introduction of a 1-moment duration stimulus, with *p*1 = 0.8, *pd*1 = 0.1, and *pd*2 = 0.02. At the moment *t*_0_, that is, before the presentation of the stimulus, all elements are in the I state; so, the activity vector is (1, 0, 0). At moment *t*_1_, *p*1 elements move to the A1 state, leaving the activity pattern in (0.2, 0.8, 0), and in moment *t*_2_, *pd*2 of these elements decay to the A2 state leaving the pattern in (0.2, 0.72, 0.08). Since the stimulus is only “on” at moment t_1_, no further elements are promoted to A1 at any other time and thus PA1 declines very rapidly. Since the rate of decay from A2 to I, *pd*2, is five times smaller than the rate of decay from A1 to A2, *pd*1, PA2 persists for a longer period. With these standard assumptions, the consequence of the presentation of a brief stimulus is a rapid and transient increase in the proportion of the elements in the A1 state, followed by an increase in the proportion of elements in the A2 sate and by a very protracted return of elements to inactivity.

Notice in Figure 1B that there is a long period after the offset of the stimulus in which a substantial proportion of elements are in the A2 state. Indeed, only at moment 250, almost all elements have decayed back to inactivity, being, thus, just then eligible for reactivation in case the stimulus was presented again at this time. This is the reason why the A2 state can be regarded as a refractory state of activity. This is illustrated in the left-hand plot of Figure 1C, which depicts the theoretical activity that would be generated if the same stimulus of Figure 1B was repeated once at an interval of 32 moments. There, it is apparent that in the second presentation, the stimulus is less effective in provoking A1 activity, which reaches a peak of about half of the size of that of the first presentation. Generally speaking, the presentation of a given stimulus may have different effects depending on the momentary distribution of elements in the three states. Since the only consequence of presenting a stimulus is through *p*1, the stimulus will have greater efficacy in provoking A1 activity the greater is the number of elements in the inactive state and the lesser in the refractory state. This feature of SOP is a quantitative rendition of priming theory which states that “when an event is pre-represented (‘primed’) in short-term memory (STM) further corresponding stimulation is rendered less effective than it otherwise would be” (Pfautz and Wagner, 1976, p. 107). In the case depicted in the figure, this priming is occasioned by previous presentations of the same stimulus, so it is referred as “self-generated priming” (Wagner, 1976, 1978).

It is clear, thus, that self-generated priming is the primary mechanism by which SOP accounts for within-session decrements or short-term habituation. Of course, this is a transient effect that disappears when sufficient time has elapsed from the last presentation of the stimulus (e.g., from one session to another). This is illustrated in the right-hand plot of Figure 1C, which reveals an almost total recovery of PA1_s_ provoked by the first presentation of the same stimulus in a separate session.

**Figure 2:**
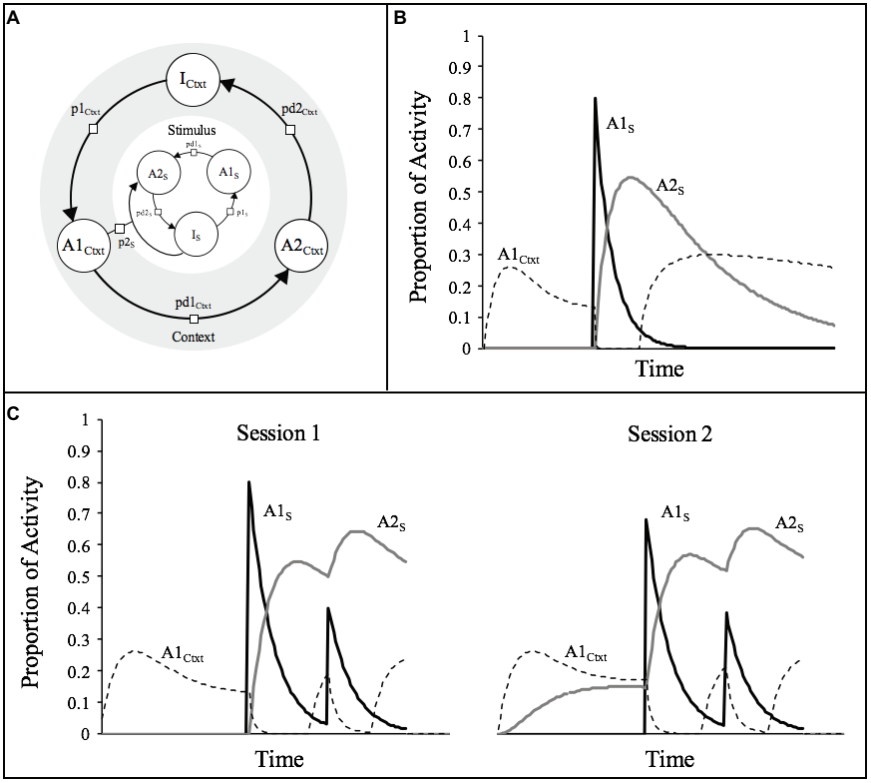
**(A)** A representation of the context-stimulus relationships assumed by SOP. **(B)** Courses of activity for a typical stimulus (s; duration = 1 moment, *p*1_S_ = 0.8, *pd*1_S_ = 0.1, *pd*2_S_ = 0.02) and the context (*p*1_Ctxt_ = 0.05; *pd*1_Ctxt_ = 0.1; *pd*2_Ctxt_ = 0.02) according to SOP rules of activity. **(C)** Simulations of the theoretical processes involved in two training sessions with two presentations of a 1-moment stimulus within the context.

Between-session effects or long-term habituation, on the other hand, require a different kind of mechanism that Wagner (1976, 1978) called “retrieval-generated priming.” In this case, the supposition was that when a stimulus is repeatedly presented in a context, the context would act as a conditioned stimulus (CS) to develop an association with the habituating stimulus, which plays the role of the unconditioned stimulus (US). As the association grows, the stimulus becomes gradually more expected in the context and thus, primed, by the context. Figure 2A sketches how SOP conceives this by assuming that both the context and the stimulus activate a respective sequence of representational nodes, and that the context, *via* its association with the stimulus, acquires the capacity to promote elements directly from I_s_ to A2_s_ *via* the variable *p*2_s_. The assumption is that *p*2_s_ is a function of the degree of primary activity of the context (A1_Ctxt_), and the strength of the association between the context and the stimulus (i.e., *p*2 = A1_Ctxt_ × V_Ctxt−s_).

According to the learning rules of SOP, changes in the net association between a CS and a US are the result of excitatory minus the inhibitory associations that develop simultaneously depending on the respective states of activity of the stimuli. The development of excitatory CS-US links, ΔV+, are assumed to be proportional to the momentary product of concurrent A1_CS_ and A1_US_ activity multiplied by an excitatory learning rate parameter, L+ (i.e., ΔV+ = L+ × PA1_CS_ × PA1_US_), whereas changes in the inhibitory CS-US connections, ΔV–, are assumed to be proportional to the momentary product of concurrent A1_CS_ and A2_US_ activity, multiplied by an inhibitory learning rate parameter, L– (i.e., ΔV– = L– × PA1_CS_ × PA1_US_). In the standard procedure to get habituation, there are several repetitions of the habituating stimulus (US) in a distinctive context (CS), which seems to comply with the conditions of SOP for strengthening the association between them.

Although this may sound straightforward, context-stimulus associations are more theoretically challenging than they appear. Vogel et al. (2018) noticed that if the context is viewed intuitively as a long duration CS with a constant value of PA1_Ctxt_ over the entire duration of the session, then SOP predicts no net association with the habituating stimulus. In this example, the net excitatory association that would be acquired by the context during the period in which the stimulus is in its A1 state of activity will be overcome by the inhibitory associations that would be provoked during the occasions in which the context is in its primary activity and the stimulus in its secondary activity during the inter-trial intervals. In order to solve this, Vogel et al. (2018) suggested that contexts should not be represented as a long uniform stimulus with a constant primary activity. They proposed that presentation of explicit cues, like the habituating stimulus, provokes systematic changes in the subject’s receptor orientation so that the processing of the context is transiently disturbed. Given the dynamics of activity of SOP, this interruption allows the context to enjoy more overlap of its A1 processing with the A1 processing of the stimulus, rather than with the later A2 processing of the stimulus. Vogel et al. posited that this is consistent with the idea that the representation of the context is very vulnerable to disruption by explicit cues.

To implement the idea of context disruption, Vogel et al. (2018) adopted the simple strategy of setting the *p*1_Ctxt_ value to zero for some period after the presentation of explicit cues. Here, we rationalize this principle in a related but different way. First, we assume that *p*1_Ctxt_ equals zero if PA1_s_ is greater than some threshold. Second, we follow Wagner’s (1981) distractor rules by stating that the decay rates from A1_Ctxt_ to A2_Ctxt_ and from A2_Ctxt_ to I_Ctxt_, respectively, are increased by the presentation of the habituating stimulus. The level of increase in these decay rates is assumed to be a function of the activity of the habituating stimulus; that is, *pd*1′_Ctxt_ = *pd*1_Ctxt_ + A1_s_/c1 and *pd*2′_Ctxt_ = *pd*2_Ctxt_ + A2_s_/c2, where *pd*1′_Ctxt_ and *pd*2′_Ctxt_ are the effective decay rates, and c1 and c2 are constant parameters of the model.

Figure 2B illustrates the effects of these assumptions on the processing of the context by simulating a situation in which the context is processed alone for some time until a 1-moment stimulus is presented. The relevant pattern of activities displayed in the figure indicates that presentation of the habituating stimulus provokes the progressive diminution of PA1_Ctxt_, which remains active for a few moments when PA1_s_ is at its maximal, but eventually gets mostly suppressed when PA2_s_ predominates. The net result of this is more excitatory learning (which is proportional to PA1_Ctxt_ × PA1_s_) than inhibitory learning (which is proportional to PA1_Ctxt_ × PA2_s_). Figure 2C presents the same simulations as those of Figure 1C, but this time we added our assumptions about the processing of the context. As can be appreciated, in session 1 there is a decrease in the amplitude of the PA1_s_ generated by the second presentation of the stimulus, which is not very different than the pattern that was described in Figure 1C. In session 2, however, the pattern is very different in that now, even in the absence of self-generated priming, the amplitude of PA1_S_ in the first presentation of the stimulus is considerably diminished. This diminution is caused by the anticipatory PA2_s_ activity provoked by the context which has developed an association with the stimulus. This between-sessions decrement is thus explained by the retrieval-generated priming announced by Wagner (1976, 1978).

## Simulations of the Characteristics of Habituation

In order to show the quantitative strength of these assumptions, in this section, we present a series of computer simulations illustrating how the model accounts for each characteristic of habituation. For this, we used the revised description of the parametric features of habituation proposed by Rankin et al. (2009).

Due to the diversity of procedures, stimuli, responses, and species underlying the corpus of research that has given rise to these characteristics, we did not attempt to mimic any specific procedure or published data in particular. Rather, we conduct all simulations with a standard procedure with minimal parametric variation from one simulation to another. Thus in all simulations, the habituating stimulus lasted 1 moment and its activation parameters were: *p*1_s_ = 0.8, *pd*1_s_ = 0.1, *pd*2_s_ = 0.02. In order to simulate high-, low-, and medium-intensity stimuli, we used three different values of *p*1_s_ = 0.8, 0.5, and 0.2. The parameters for activation of the context were identical to those of the habituating stimulus excepting for a lower *p*1 value. That is, *p*1_Ctxt_ = 0.05, *pd*1_Ctxt_ = 0.1, and *pd*2_Ctxt_ = 0.02. The context was turned on at the first moment of each simulation and stayed on according to its *p*1, *pd*1, and *pd*2 values unless the habituating stimulus is presented (which occurred at moment 60 of the simulation). Specifically, if PA1_s_ > 0.07 then, *p*1_Ctxt_ = 0. The presentation of the habituating stimulus also increases the decay parameters of the context to *pd*1′_Ctxt_ = 0.1 + PA1_S_/2 and *pd*2′_Ctxt_ = 0.02 + PA2_S_/10.

To simulate the transition from one session to another, all activity was set to zero at the end of the session. Only the associative values of the context were carried on from one session to the next. For the simulations, we used the software Stella® Architect (Isee systems; Lebanon, NH, United States).

### Simple Within-Session Effects

**Figure 3:**
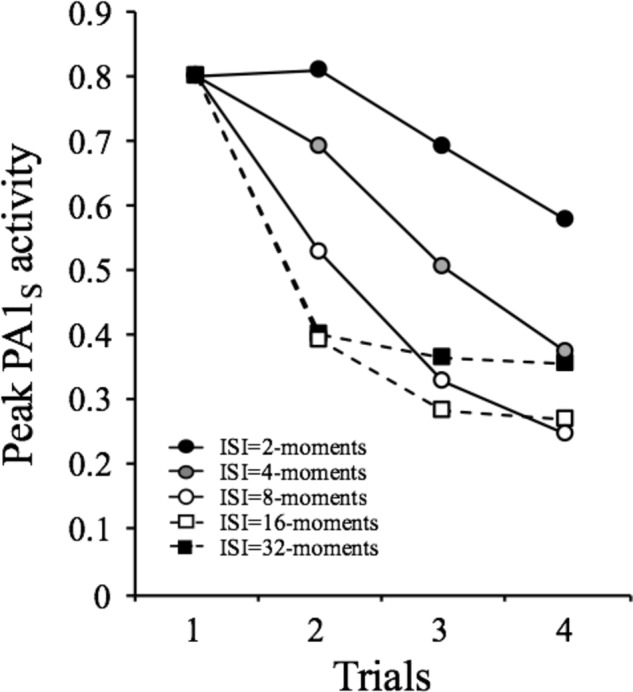
Simulated peak PA1_S_ activity over 4 presentations of a 1-moment stimulus at inter-stimulus intervals ranging from 2 to 32 moments.

The simulations described in Figures 1C and 2C attempted to make clear that decrements in responding that occur within a session are mainly explained by self-generated priming. In order to illustrate the generality of this effect, we conducted a series of computer simulations in which a 1-moment duration stimulus was repeated four times at inter-stimulus intervals (ISI) of 2-, 4-, 8-, 16-, or 32-moments in a single session. The results are depicted in Figure 3 in terms of the peak PA1_s_ activity provoked by each presentation of the stimulus. The general pattern is that in each occasion, the stimulus becomes less effective in provoking its A1_s_ activity than in the previous occasions. The decrease in most cases approximates an exponential function except for the shortest ISI, in which there is transient facilitation. According to SOP, this facilitation occurs only when the two presentations of the stimulus are sufficiently close in time to produce a summation of PA1_s_. Beyond the cases of very short ISI (2 and 4 moments), the model predicts that the longer the interval, the less pronounced is the decrement at the end of the session.

This pattern approximates well to the first empirical feature of habituation listed by Thompson and Spencer (1966) and reviewed by Rankin et al. (2009) as follows:

“Repeated application of a stimulus results in a progressive decrease in some parameter of a response to an asymptotic level. This change may include decreases in frequency and/or magnitude of the response. In many cases, the decrement is exponential, but it may also be linear; in addition, a response may show facilitation prior to decrementing because of (or presumably derived from) a simultaneous process of sensitization.” (Characteristic #1, p. 136).

### Spontaneous Recovery and Long-Term Habituation

Here, we analyze SOP’s account of several related facts of habituation listed by Rankin et al. (2009). One refers to the fact that “if the stimulus is withheld after response decrement, the response recovers at least partially over the observation time (‘spontaneous recovery’).” (Characteristic #2, p. 136). Another is the observation that “Some stimulus repetition protocols may result in properties of the response decrement (e.g., more rapid rehabituation than baseline, smaller initial responses than baseline, smaller mean responses than baseline, less frequent responses than baseline) that last hours, days or weeks. This persistence of aspects of habituation is termed long-term habituation.” (Characteristic #10, p. 137).

According to the model, the self-generated priming effects that occur within a session of habituation tend to disappear with the passage of time. This gives rise to the prediction of “spontaneous” recovery of the response from one session to another. The retrieval-generated priming caused by the context, however, does not depend on temporal factors but on the use of the same context in the two sessions. This gives rise to the prediction of a long-term decrement from one session to the next. Thus, in principle, it seems relatively straightforward to conclude that the model predicts a partial recovery of responding from session to session, which would result from the combination of the natural termination of self-generated priming and the persistence of retrieval-generated priming.

Another characteristic of spontaneous recovery that is consistent with this analysis is that “after multiple series of stimulus repetitions and spontaneous recoveries, the response decrement becomes successively more rapid and/or more pronounced (this phenomenon can be called potentiation of habituation).” (Characteristic #3, p. 136). According to SOP, every repetition of the stimulus will lead to an increase in the association between the context and the cue, so more decrement and less spontaneous recovery are expected over extensive training.

**Figure 4:**
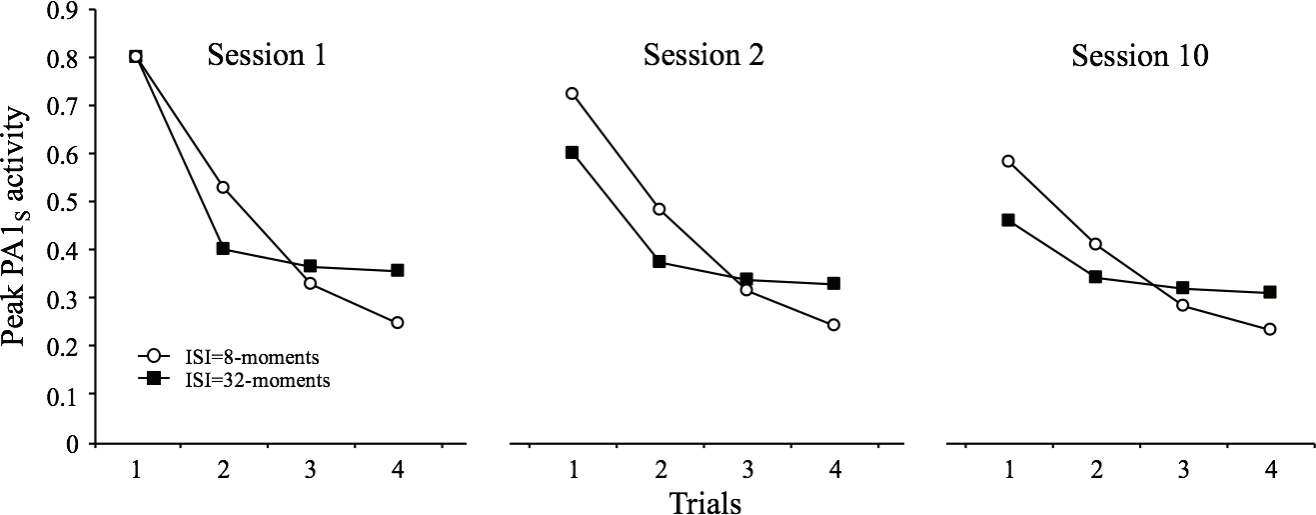
Simulated peak PA1_S_ activity over 4 presentations of a 1-moment stimulus at inter-stimulus intervals of 8 and 32 moments. The figure contrasts the courses of within-session decrements and spontaneous recovery for the two ISIs during the first, second, and tenth sessions of training.

The simulations presented in Figure 4 illustrate how SOP accounts for all the characteristics described above. The simulation involved four presentations of the stimulus at intervals of 8 and 32 moments in each of three identical sessions. The results are clear for the two conditions: there is a partial recovery in the PA1_s_ from the last trial of one session to the first trial of the next (spontaneous recovery), and there is a diminution in the degree of spontaneous recovery in session 10 relative to session 2 (potentiation of habituation).

The data displayed in the figure also allow for the analysis of a further characteristic (#4), which states that “Other things being equal, more frequent stimulation results in more rapid and/or more pronounced response decrement, and more rapid spontaneous recovery (if the decrement has reached asymptotic levels).” (Rankin et al., 2009, p. 136). This is seen in the figure by comparing the within- and between-session decrements for the two simulated ISIs. That is, there are more within-session decrement and more spontaneous recovery for the 8-moments ISI than for the 32-moments ISI.

**Figure 5:**
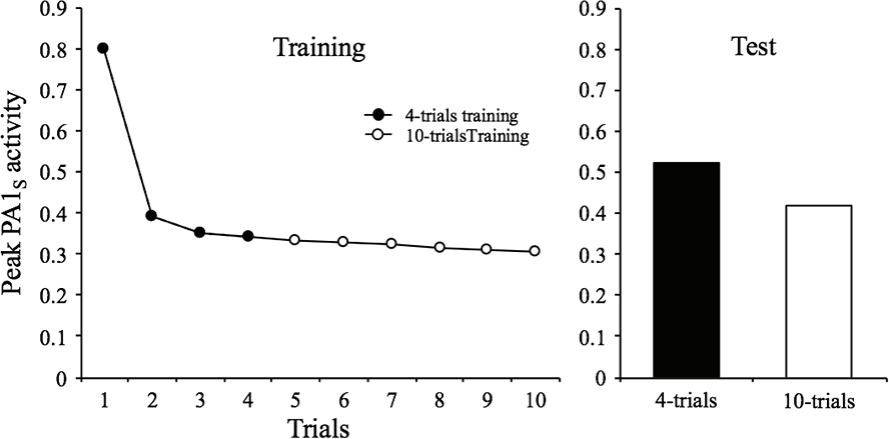
Simulated peak PA1_S_ activity over 4 or 10 presentations of a 1-moment stimulus at a 32-moments inter-stimulus interval. The bar graph depicts the peak PA1_S_ activity in a single spontaneous recovery test-trial for the two training conditions.

Finally, there is a further characteristic that can be embraced by the context-stimulus association. This property is listed as the sixth characteristic and described by Rankin et al. (2009) as: “The effects of repeated stimulation may continue to accumulate even after the response has reached an asymptotic level (which may or may not be zero, or no response). This effect of stimulation beyond asymptotic levels can alter subsequent behavior, for example, by delaying the onset of spontaneous recovery.” (p. 137). SOP explains this phenomenon, also known as “below-zero habituation,” by appealing to the fact that once the level of PA1s has reached a low asymptotic value within a session, further training can increase *V*_Ctxt-S_ with no major observable effect in this session but that will be apparent in a spontaneous recovery test in another session. Figure 5 illustrates this by displaying the result of a computer simulation in which the habituating stimulus was presented either 4 or 10 times at a 32-moments interval. As can be seen, the level of PA1_s_ in the fourth trial of the short training condition is almost identical to the tenth trial of the extended training condition. Nonetheless, in the tests conducted in session 2, there is more decrement in the extended condition relative to the short-training condition.

### Stimulus Properties

**Figure 6:**
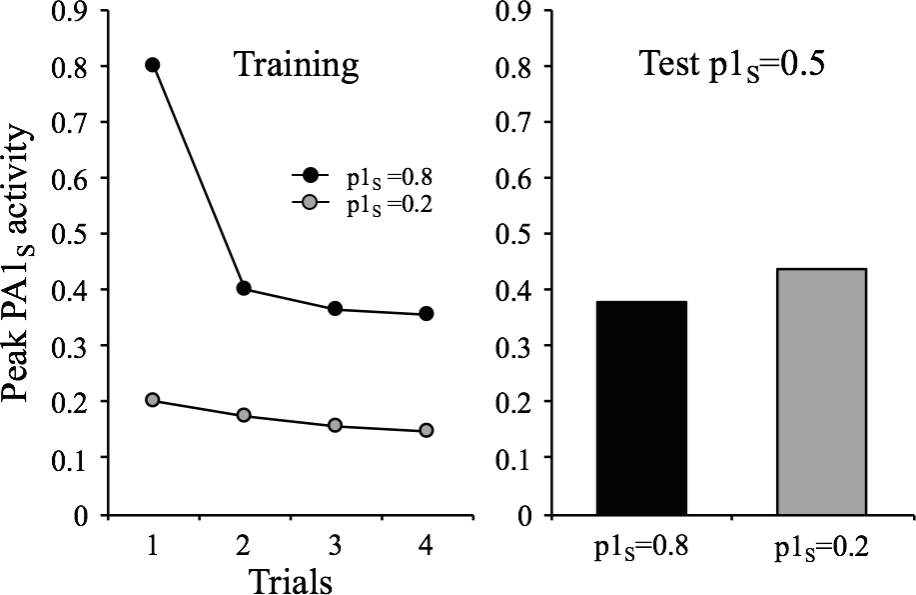
Simulated peak PA1_S_ activity over 4 presentations of a 1-moment duration stimulus at a 32-moments ISI, under two intensity conditions: high intensity (*p*1_s_ = 0.8) and a low intensity (*p*1_s_ = 0.2). The bar graph depicts the peak PA1_S_ activity in a single spontaneous recovery test-trial for the two training conditions tested with a common *p*1_s_ = 0.5.

There are two characteristics of habituation listed by Rankin et al. (2009) that can be explained by some features of the habituating stimulus. One says that “within a stimulus modality, the less intense the stimulus, the more rapid and/or more pronounced the behavioral response decrement. Very intense stimuli may yield no significant observable response decrement.” (Characteristic #5, p. 137). As mentioned before, the parameter *p*1 in the model can be assumed to represent the intensity of the stimulus. In terms of the model, *p*1 influences two relevant processes: performance and learning. That is, the higher the value of *p*1, the higher is the response and the faster is learning. The result of this is shown in Figure 6, which depicts the results of a simulation in which the habituating stimulus was presented four times with *p*1_s_ values of 0.2 and 0.8 and then tested in a subsequent session with a common *p*1_s_ of 0.5. As can be appreciated, the *p*1_s_ = 0.2 condition exhibited more within-session decrements, but less between-session decrements than the *p*1_s_ = 0.8 condition. Of course, the within-session effect is a mere performance effect (i.e., less responding to lower *p*1_s_) while the between-session effect is a reflection of differential context-stimulus learning (more learning, and therefore, less responding for higher *p*1_s_).

**Figure 7:**
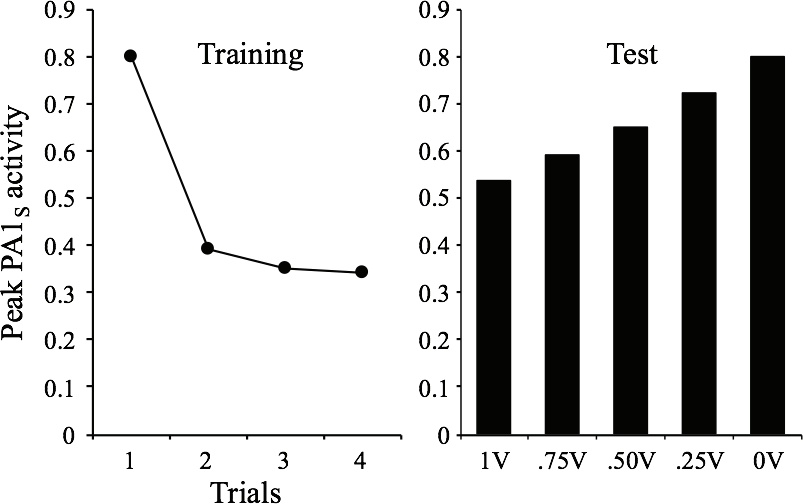
Simulated peak PA1_S_ activity over 4 presentations of a 1-moment stimulus at a 32-moments inter-stimulus interval. The bar graph depicts the peak PA1_S_ activity in a single spontaneous recovery test-trial for stimuli that received a rate of 1, 0.75, 0.5, 0.25, and 0 of generalized V from the habituated stimulus.

Another property is stimulus generalization, which is described by Rankin et al. (2009) as follows: “Within the same stimulus modality, the response decrement shows some stimulus specificity. To test for stimulus specificity/stimulus generalization, a second, novel stimulus is presented, and a comparison is made between the changes in the responses to the habituated stimulus and the novel stimulus.” (Characteristic #7, p. 137). This property does not pose a special theoretical difficulty for any theory of stimulus processing. To account for it, it is sufficient to assume some generalization gradients for stimulus variation. For the sake of simplicity, here we just make the simple assumption that the context-stimulus association is generalized from one stimulus to another as a function of their similarity. Figure 7 presents the results of these assumptions showing that after training a given stimulus for four trials at an interval of 32 moments, the peak PA1_s_ values are proportional to the assumed percent of generalization of *V*_Ctxt-stimulus_.

### Dishabituation

There are two further characteristics listed by Rankin et al. (2009) that refer to the effects of the presentation of a novel stimulus or distractor in the middle of a sequence of presentations of the habituating stimulus. The first states that a “presentation of a different stimulus results in an increase of the decremented response to the original stimulus. This phenomenon is termed ‘dishabituation.’” (Characteristic #8, p. 137). The second says that “upon repeated application of the dishabituating stimulus, the amount of dishabituation produced decreases (this phenomenon can be called habituation of dishabituation).” (Characteristic #9, p. 137).

**Figure 8:**
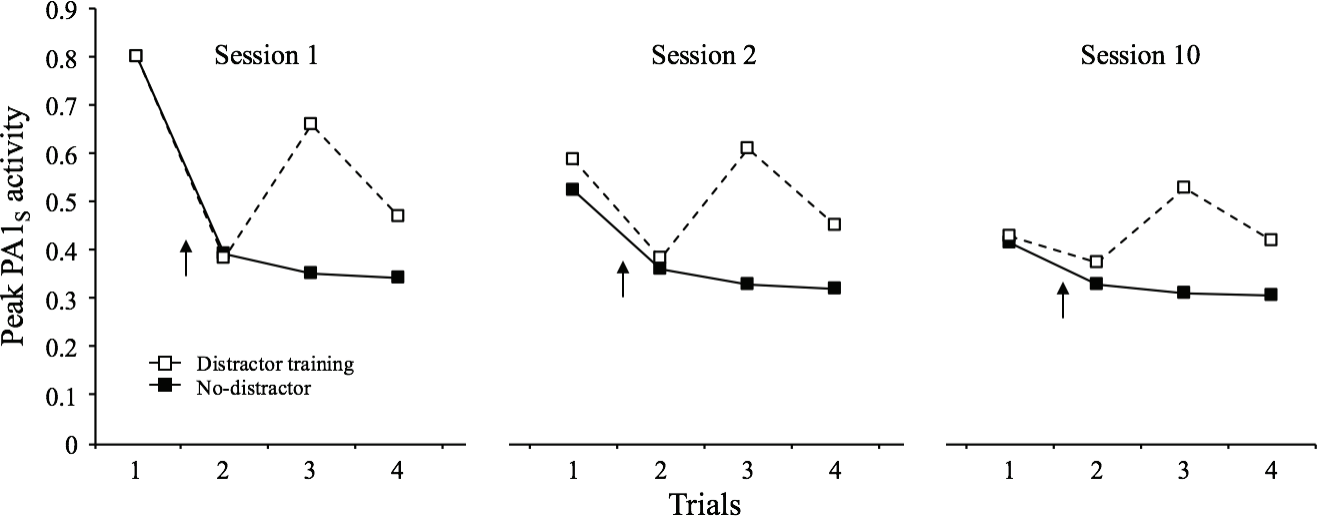
Simulated peak PA1_S_ activity over 4 presentations of a 1-moment stimulus at a 32-moments inter-stimulus interval over 3 training sessions. In the distractor-training condition, a novel 1-moment stimulus (*p*1 = 0.8; *pd*1 = 0.1, and *pd*2 = 0.02) was presented between trial 1 and 2 of each session, while in the other condition there was no such a stimulus in any trial The arrow indicates the distractor location in each case.

As described above, SOP provides with a set of “distractor rules” by which the presentation of a novel stimulus shortly before a target stimulus causes increments in the decay rates of the target stimulus (*pd*1 and *pd*2). These increments are proportional to the degree of primary and secondary activity of the distractor. To exemplify this, Figure 8 depicts the results of a simulation in which the habituating stimulus was presented four times at an interval of 32 moments in each of three identical sessions. In one condition, a distractor was presented between trial 1 and 2 of each session, while in the other condition, there was no distractor in any trial. The results indicate that the distractor provoked an increase in PA1_s_ in the subsequent trials relative to the non-distractor condition. Although this effect was notorious in all three sessions, it became progressively less robust over sessions (habituation of dishabituation). The last effect is due, in part, to the fact that as the distractor itself is repeated, it becomes associated with the context and thus rendered less effective.

## Final Commentaries

In this paper, we showed how the major features of the historically classic phenomenon of habituation can be modeled by the quantitative instantiation of the principles embedded in an already classic theory of stimulus processing, the SOP model (Wagner, 1981). Although this may sound outdated, one reason for bringing such issues here is that theorizing in this field has been relatively neglected, especially in the domain of quantitative modeling.

In the present exercise, we preferred to keep the analysis as simple as possible for expository reasons. But, of course, we must recognize that our simulations of the 10 characteristics of habituation do not exhaust the empirical wealth of the field. Thus, and before concluding, let us make a brief reference to a couple of issues that can be taken forward in future theoretical analyses.

The first refers to the fact that every stimulus evokes several distinct types of responses. These responses may have very different topographies and be differentially susceptible to habituation. In SOP, this difference can be modeled by variations in the parameters of activation. Wagner and Brandon (1989) suggested, for instance, that emotional responses to an aversive stimulus may be represented by more delayed decay processes (i.e., smaller *pd*1_s_ and *pd*2_s_) than the sensory response to the same stimulus. With this parametric variation, one can expect, in principle, that emotional responses will be associated more rapidly with the context than sensory responses. This might explain, in part, the fact that different measures of habituation can show differential context specificity (e.g., Jordan et al., 2000; Pinto et al., 2014).

Furthermore, according to SOP, the repetition of an aversive stimulus, apart from leading to habituation, can also result in the conditioning of emotional responses that potentiate the response to the habituating stimulus itself. Wagner and Vogel (2010) proposed that emotive sensitization competes with sensory habituation in complex ways, such that habituation might be obscured by potentiating effects, presumably reflecting the contribution of an emotional response controlled by the same context that controls habituation. The co-existence of several types of interacting associations between the context and the stimulus is conceptually consistent with SOP. This analysis must be complemented, however, with sufficient empirical studies that succeed in dissociating the response-potentiating from the response-diminishing effects of stimulus repetition (Ponce et al., 2011; Ponce et al., 2015).

There is one further aspect of SOP that was left untreated in the present analysis of habituation. Wagner (1981) proposed that the response to the stimulus is a function of PA1 and PA2 of the stimulus; that is, *R* = f × (wl × PAl_S_ + w2 × PA2_S_), where wl and w2 are linear weighting factors, and f is a mapping function appropriate to the response measure of interest. As Donegan and Wagner (1987) suggested, this equation provides for at least three options that have differential impact on the course of habituation. One is assuming a very low value of w2, say zero, as we did in this paper. In this case, the response would depend entirely on PA1_s_, with PA2_s_ contributing only indirectly *via* its priming effect on PA1_s_. In our simulations, we have adopted this tactic because it seems to represent better the predominant types of responses that were used for the definition of the 10 characteristics of habituation (e.g., limb flexion in the spinal cat and startle response in rats).

Another possibility is to adopt a sizable and negative value for w2. In this case, the conditioned and unconditioned secondary activity is subtracted from the primary activity to produce the response. Here, both the negative contribution of PA2_s_ to the response and its priming on PA1_s_ would act in a synergic way to diminish the primary response to the stimulus. The use of w1 and w2 with opposite signs may be particularly advised when there are empirical reasons to believe that the response to the habituating stimulus shows a secondary response that opposes the primary response as it has been frequently reported with pharmacological stimuli (e.g., Siegel, 2005).

The third theoretical alternative is to assume that w2 is substantial and positive. Here, PA2_s_ would have two opposite effects on the response: an augmentative effect through summation with PA1_s_ and a diminutive effect through priming. In this more complex scenario, it would be expected to observe less behavioral habituation than in the former cases. Although it may be difficult to assess this possibility with the standard habituation procedures, it is consistent with reports of enhanced performance in some perceptual tasks when the target stimulus is preceded by the same or an associated stimulus (e.g., Posner and Snyder, 1975; Kristjansson and Campana, 2010; Henson et al., 2014).

It may be seen that, despite its complexity, the SOP model is quite well articulated and as such, it seems to be uniquely equipped to encourage further theoretical and empirical work beyond the 10 features of habitation and for a range of very distinct stimulus–response systems. It should be said also that the explanatory scope of the model is not restricted to habituation. Its usefulness has been demonstrated in a variety of phenomena, mainly in the domain of associative learning, such as occasion setting (Wagner and Brandon, 2001; Vogel et al., 2017), timing (Vogel et al., 2003), divergence of response measures (Wagner and Brandon, 1989), trial spacing (Sunsay and Bouton, 2008), cue competition (Mazur and Wagner, 1982; Vogel et al., 2015), causal learning (Dickinson and Burke, 1996; Aitken and Dickinson, 2005), mediated conditioning (Dwyer et al., 1998; Pearce and Bouton, 2001), latent inhibition (Honey and Hall, 1989), and object recognition (Honey and Good, 2000; Robinson and Bonardi, 2015).

In concluding, let us make a personal statement. This paper was prepared in response to the call for papers to be published in a special issue of Frontiers in Psychology on “Research in emotion and learning: Contributions from Latin America.” The authors of this article work in Talca, Chile, and we were all tremendously influenced by Allan R. Wagner. His influence was not just intellectual but also took the form of concrete contributions to the setting up of our laboratory for the study of learning in Chile. Allan had accepted to write this paper in collaboration with us. He agreed with the general approach of the paper and with the novel instantiation for context learning, but he passed away before any of the work was completed.

## Author Contributions

EV and YU-B contributed to the conception of the study; FP, YU-B, and SB conducted the simulations and organized the database; and EV wrote the first draft of the manuscript. All authors contributed to manuscript revision, read and approved the submitted version.

### Conflict of Interest Statement

The authors declare that the research was conducted in the absence of any commercial or financial relationships that could be construed as a potential conflict of interest.
